# Bioelectrical Impedance Vector Analysis Discriminates Aerobic Power in Futsal Players: The Role of Body Composition

**DOI:** 10.3390/biology11040505

**Published:** 2022-03-25

**Authors:** Catarina N. Matias, Francesco Campa, Giuseppe Cerullo, Giuseppe D’Antona, Rita Giro, João Faleiro, Joana F. Reis, Cristina P. Monteiro, Maria J. Valamatos, Filipe J. Teixeira

**Affiliations:** 1 Bettery Life Lab, Innovation Direction, Bettery S.A., 2740-262 Lisboa, Portugal; katarina_matias@hotmail.com (C.N.M.); rita.giro@betterylife.com (R.G.); joaof264@gmail.com (J.F.); filipe.teixeira@betterylife.com (F.J.T.); 2 CIDEFES—Universidade Lusófona, 1749-024 Lisboa, Portugal; 3 Department for Life Quality Studies, University of Bologna, 47921 Rimini, Italy; 4 Department of Movement Sciences and Wellbeing, University of Naples Parthenope, 80133 Naples, Italy; giuseppe.cerullo001@studenti.uniparthenope.it; 5 Centro di Ricerca Interdipartimentale nelle Attività Motorie e Sportive (CRIAMS)—Sport Medicine Centre, University of Pavia, 27058 Voghera, Italy; giuseppe.dantona@unipv.it; 6 AC Oulu Football Club, 90100 Oulu, Finland; 7 Interdisciplinary Center for the Study of Human Performance (CIPER), Faculdade de Motricidade Humana, Universidade de Lisboa, 1495-761 Cruz-Quebrada, Portugal; joanareis@fmh.ulisboa.pt (J.F.R.); cmonteiro@fmh.ulisboa.pt (C.P.M.); mjvalamatos@fmh.ulisboa.pt (M.J.V.); 8 Laboratory of Physiology and Biochemistry of Exercise, Faculdade de Motricidade Humana, Universidade de Lisboa, 1495-761 Cruz-Quebrada, Portugal; 9 Neuromuscular Research Lab, Faculdade Motricidade Humana, Universidade Lisboa, 1495-761 Cruz-Quebrada, Portugal; 10 Atlântica, Instituto Universitário, Fábrica da Pólvora de Barcarena, 2730-036 Barcarena, Portugal

**Keywords:** athletes, BIA, BIVA, fat mass, phase angle, performance, sports practice, VO_2max_

## Abstract

**Simple Summary:**

Bioelectrical and body composition proprieties are linked to health status and physical performance. The bioelectrical impedance vector analysis (BIVA) is a valid approach for qualitatively and timely assessing body components in sports practice. However, it is still unclear, if bioimpedance vector patterns can provide relevant information regarding the aerobic power. For the first time, this study shows that BIVA is useful in discriminating professional futsal players according to their VO_2max_. In addition, fat mass and bioelectrical phase angle were identified as valid predictors for aerobic power.

**Abstract:**

Aims: The present study aimed to assess the ability of bioelectrical impedance vector analysis (BIVA) in discriminating fitness levels in futsal players, exploring the association of body composition and bioelectrical parameters with aerobic power. Methods: Forty-eight professional futsal players (age 23.8 ± 5.3 years) were involved in a cross-sectional study during their pre-season phase. Fat mass (FM) and muscle mass were determined by dual-energy X-ray absorptiometry. VO_2max_ was obtained by indirect calorimetry through a graded exercise test performed on a treadmill. Bioelectrical resistance (R), reactance (Xc), and phase angle (PhA) were directly measured using a foot-to-hand bioimpedance technology at a 50 kHz frequency. Bioelectric R and Xc were standardized for the participants’ height and used to plot the bioimpedance vector in the R-Xc graph according to the BIVA approach. Results: The participants divided into groups of VO_2max_ limited by tertiles showed significant differences in mean vector position in the R-Xc graph (*p* < 0.001), where a higher VO_2max_ resulted in a longer vector and upper positioning. FM, muscle mass, and PhA differed (*p* < 0.01) among the athletes grouped by tertiles of VO_2max_, where athletes with a greater aerobic power showed a lower percentage of FM and a higher percentage of muscle mass and PhA. FM and PhA were associated with VO_2max_ (FM: r = −0.658, *p* < 0.001; PhA: r = 0.493, *p* < 0.001). These relationships remained significant after adjusting for age and body mass (FM: ß = −0.335, *p* = 0.046; PhA: ß = 0.351, *p* = 0.003). Conclusions: Bioelectrical impedance vectors positioned on the lower pole of the R-Xc graph identified futsal players with a lower VO_2max_, while longer vectors corresponded to a greater aerobic power. Additionally, PhA, that describes the vector direction, was positively associated with VO_2max_, while a higher FM negatively affected VO_2max_ in the futsal players. BIVA and PhA evaluation may represent a valid support for screening the aerobic fitness level in professional futsal players, when more sophisticated assessment methods are not available.

## 1. Introduction

Futsal is a 5-a-side indoor adaptation of soccer that is officially recognized by the soccer’s international governing body (FIFA). Despite increasing in popularity around the world, with over 12 million athletes participating in over 100 nations, the body of literature related to futsal is still scarce. The futsal game is played between two teams, each with five players: one goalkeeper and four outfielders, usually known as defenders, right or left-wingers, and pivots. Competitive matches include periods of intermittent and high-intensity activity that require significant physical, technical, and tactical effort [1,2]. The highest metabolic contribution is provided by the aerobic pathway as recently reported in professional futsal players during a simulated futsal game [3]. The maximal oxygen uptake (VO_2max_) is among the most important determinants for endurance performance, as well as mortality in the general population [4,5]. In this regard, VO_2max_ is considered the gold standard for assessing aerobic fitness. However, it is possible to observe a lack of scientific data related to aerobic capacity of futsal players. The VO_2max_ mirrors the maximum amount of oxygen an individual can use to support the oxidative production of energy [6]. The most common and reliable method to assess VO_2max_ is the cardiopulmonary exercise test, recognized a valuable measure not only to assess the functional status and oxygen availability during exercise training but also to discriminate cardiorespiratory, pulmonary and musculoskeletal function at the same average oxygen consumption [7]. For the first time, Alvarez et al. [8] showed that high values of VO_2max_ were essential for futsal athletes at the professional level, suggesting the relevance of aerobic power in futsal [8]. Subsequently, other authors confirmed that VO_2max_ may be assumed as a discriminative physiological parameter in futsal played at recreational or professional levels [9,10,11]. Nevertheless, differences in this regard may be a result of genetic factors [4], players role [8], training load [11] or body composition [12]. In general, increased body fat is associated with decreased value of VO_2max_, while FFM is positively correlated with VO_2max_ [13,14]. Futsal players usually display a low percentage of fat (~15%) with a higher lean mass in high-level players [12]. Taken together, these data might suggest a close relationship between body composition and aerobic power in this sportive population.

Body composition parameters can be accurately determined using laboratory techniques and procedures [15]. To date, the state-of-art method for determining body fat is identified in a specific formula proposed by Wang et al. [15], which requires the assessment of bone mineral content by dual-energy X-ray absorptiometry (DXA), total body water (TBW) by deuterium dilution, and body volume by air displacement plethysmography. In addition to bone mineral content, for which it is considered the gold standard, DXA allows for accurate estimations of lean soft and muscle tissues [16]. However, these procedures are difficult to use in the practical context due to their high cost and the need for specialized personnel [17]. The bioelectrical impedance analysis (BIA) has been suggested as an accurate method for assessing body composition in athletes, provided that specific procedures are used [17]. In particular, single frequency BIA provides body composition estimations with a good agreement respect to the aforementioned reference methods [17,18]. However, the most innovative use of BIA consists in the evaluation of the raw bioelectrical parameters through the vector analysis (BIVA) [18,19]. Indeed, the bioimpedance can be considered as the bivariate result of the bioelectrical resistance (R) and reactance (Xc) and graphically represented as a vector into a graph [18,20]. Particularly, the direction of the vector is determinate by the phase angle (PhA), which can be directly calculated as the arctangent of Xc and R [18]. In BIVA, the vector length is representative of the TBW, where a higher fluid content corresponds to long vectors. Additionally, PhA is positively associated with the intracellular-to-extracellular water (ICW/ECW) ratio [21] and can be evaluated when more sophisticate methods for determining body fluids are not available. The evaluation of BIVA patterns represents a qualitative examination of body composition and avoids the use of prediction equations. Recent literature reviews have shown how BIVA is able to discriminate different body morphologies and identify adaptations related to sports performance in athletes [18,19,22]. 

To the best of our knowledge, the ability of BIVA and the role of the bioelectrical properties in discriminating aerobic power in futsal players is still unexplored. The use of BIVA could serve as a practical tool suited for nutritionists and futsal coaches and team staff in obtaining information on VO_2max_. Therefore, the present study aimed to investigate the ability of BIVA in defining aerobic power in futsal players, clarifying the role of body composition in the VO_2max_ prediction. Our hypothesis was that BIVA would be informative for aerobic performance and that body composition would be highly correlated with VO_2max_.

## 2. Materials and Methods

### 2.1. Study Design and Participants

In line with previous studies to evaluate the ability of BIVA in discriminating physical characteristics, by grouping the participants into tertiles of VO_2max_, a cross sectional study design is presented. DXA was used as a support to obtain additionally information related to body composition. The sample size was assessed a priori using GPower (version 3.1, Dusseldorf, Germany). It was calculated considering a large effect size (appropriate for calculating effect size within a multiple regression model with continuous independent and dependent variables), with a 5% type I error, 85% power, and an α level = 0.05, which resulted in the appropriate sample size for conducting this study. The final sample was comprised of both elite and sub elite players (age 23.8 ± 5.3 years). The elite players (n = 16) participated in the Major Portuguese National Futsal League “LIGA PLACARD” and had a sport-specific training frequency of 5 sessions per week and a game frequency of 1 per week. The sub elite players (n = 32) participated in either the second (n = 16) or third (n = 16) Portuguese National Futsal League and they endured 3 training sessions of ~1.5 h per week plus a weekend game. This investigation was approved by the Faculty of Human Kinetics Institutional Review Board (approval number 37/2021) and conformed to all standards of human research set out in the declaration of Helsinki. After a detailed explanation of the procedures, the participants signed an informed consent. 

### 2.2. Procedures

All evaluations of the participants were performed early in the morning (7.00 a.m.) after a 12-h fast and without consumption of alcohol, caffeine/stimulant beverages and at least 12 h from the last exercise session. Body composition assessments were performed at fast, while the cardiopulmonary exercise test (CPET) to access VO_2max_ was made in a fed state, with a meal replacement bar (nutritional composition: 231 kcal, 14 g of fat, 12 g of carbohydrate and 13 g of protein) being provided prior to testing.

**Figure 1:**
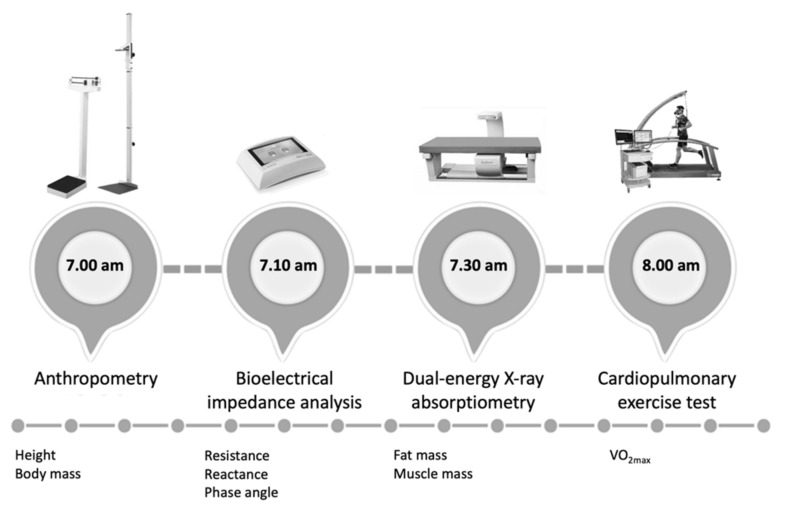
Timeline of stations performed by the futsal players involved in the study.

All measurements were performed in the pre-season period following the timeline depicted in Figure 1.

The participants had their weight and height measured wearing minimal clothes and without shoes to the nearest 0.1 kg and 0.1 cm, respectively with a scale and a stadiometer (Seca, Hamburg, Germany). Body composition was determined through two methodologies:

(a) Whole-body DXA scan (Horizon Wi, Waltham, MA, USA) according to the procedures recommended by the manufacturer. The DXA measurements included whole-body estimations of absolute and percentage of fat mass (FM, kg and %) and lean soft tissue, from which muscle mass was calculated using the Kim’s formula [16].
(b) Whole-body BIA using a single frequency of 50 kHz device (BIA 101 BIVA® PRO, Akern Systems, Firenze, Italy). After cleaning the skin with isotropy alcohol, four low intrinsic impedance adhesive electrodes (Biatrodes Akern Srl, Firenze, Italy) were placed on the hands back and other four electrodes on the neck of the corresponding feet, according to the guidelines for athletes [18]. From the raw data R and Xc, PhA was calculated as the arctangent of Xc/R × 180/π. BIVA was applied standardizing R and Xc for the subjects’ stature in meters.

After body composition assessments, a maximal incremental test with expired gas analysis (Quark, Cosmed, Italy) was performed on a motor-driven treadmill. After 3 min of warm-up at 5 km∙h^−1^, participants began the test at 6 km∙h^−1^ and 2% grade. Each minute the speed increased 1 km∙h^−1^ until volitional exhaustion, so that fatigue would be induced within 8–12 min [23]. The highest 30 s average of VO_2_ values reached during the exercise phase of the incremental test were considered to be the VO_2max_. At the end of the test, all subjects met at least two of the following criteria: respiratory quotient greater than 1.10; heart rate equal to or greater than 95% of predicted maximal HR; and increments in VO_2_ below 2 mL∙kg^−1^∙min^−1^ despite an increase in speed [24].

### 2.3. Statistical Analysis

Data were analyzed with IBM SPSS Statistics, version 24.0 (IBM Corp., Armonk, NY, USA). Descriptive statistics (mean ± standard deviation) were calculated for all measurements. To verify the normality of the data, the Shapiro-Wilk test was applied. The participants were divided into groups limited by tertiles of VO_2max_ [first tertile (T1), second tertile (T2) and third tertile (T3)] and a one-way ANOVA was performed to evaluate differences in body composition. When a significant F ratio was obtained, the Bonferroni post hoc test was used to assess the differences between the three groups, setting the significance at *p* < 0.016. The unpaired-sample Hotelling’s T^2^ test was used to compare the mean impedance vectors among the participants grouped into tertiles. Mahalanobis distance (D^2^), which represents a multivariate measure of effect and a multivariate measure of distance, was calculated to determine the magnitude of changes in the mean group vectors. D^2^ was interpreted according to the following Stevens’s [25] guidelines: 0.25–0.49: small; 0.5–0.99; ≥1: large. Bivariate correlations were performed in preliminary analysis and multiple regression analysis was used to determine if body components were significant predictors of VO_2max_ after correcting for age and body mass.

## 3. Results

No difference for age among the three groups was found (F = 0.41, *p* = 0.661). Table 1 shows the descriptive characteristics for the athletes divided according to tertiles of VO_2max_. The athletes with lower VO_2max_ (T1) showed higher body mass and absolute (kg) and relative (%) FM than the other groups. Additionally, athletes included into the T1 reported lower percentage of muscle mass, Xc/H, and PhA than the T3 group. 

**Figure 2:**
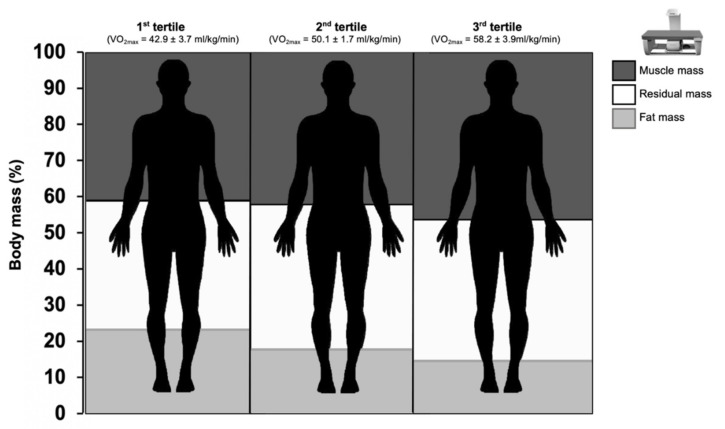
Body composition assessed with DXA according to the three-compartments tissue model in the futsal players grouped by tertiles of VO_2max_.

Figure 2 shows the relative amount of FM and muscle mass according with the three—compartments tissue model.

**Figure 3:**
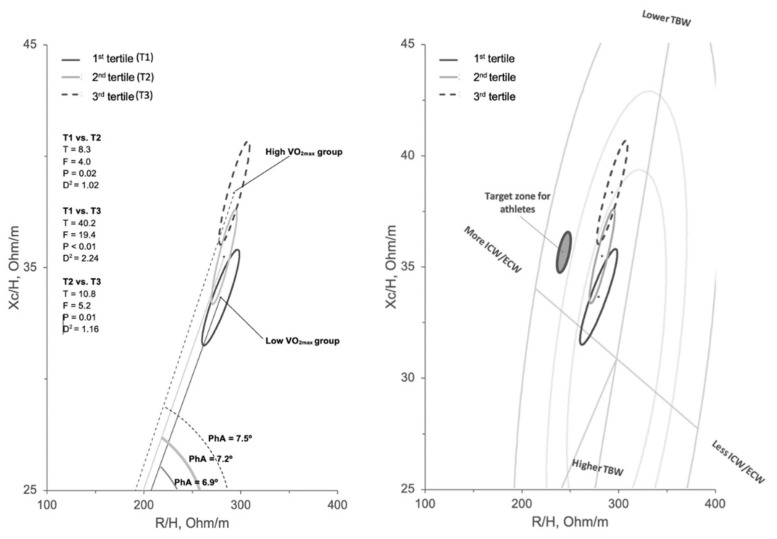
On the left, mean impedance vectors with their 95% confidence ellipses for VO_2max_ tertiles and Hotelling’s T^2^ test results; vector direction defined by phase angle (PhA) is also shown. On the right side, the target zone for athletes [26] is reported and the tolerance ellipses for the general population [27] depicted in the background.

Figure 3 shows the BIVA patterns for the three groups. The athletes with higher VO_2max_ showed a mean vector displaced in the higher part of the graph, out of the 50th percentile of the tolerance ellipses of the general population [23]. The T2 and T3 groups fell into the 50th percentile of the R-Xc graph. Significant bivariate differences in R/H and Xc/H among groups were found, as shown in Figure 1. Figure 1 the mean vectors with their 95% confidence ellipses are compared with the target zone proposed for the athletic population. The T1 and T3 groups showed different vector directions, as highlighted by the difference in PhA among these two groups.

**Figure 4:**
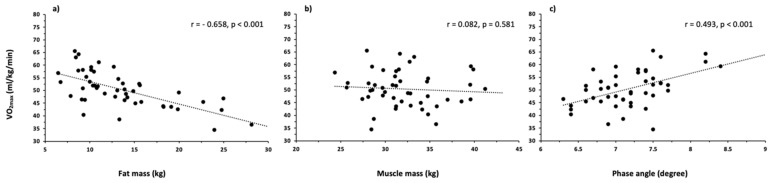
Correlation between fat mass (panel (**a**)) and muscle mass (panel (**b**)) phase angle (panel (**c**)) with VO_2max_ in the futsal players.

VO_2max_ was negatively correlated with FM (r = −0.658, *p* < 0.001), while positively associated with PhA (r = 0.493, *p* < 0.001), as showed in Figure 4. No association between muscle mass and VO_2max_ was found (r = −0.082, *p* = 0.581) (Figure 4).

The results of the multivariate regression analysis showed that FM% and PhA remained significant predictor even when adjusted for age and body mass, as reported in Table 2. 

## 4. Discussion

The present study aimed to assess the ability of BIVA in discriminating the variability in VO_2max_ and the role of body composition in determining VO_2max_ in elite and sub-elite futsal players. When the futsal players were grouped in tertiles for VO_2max_, they showed specific positions on the R-Xc graph, suggesting different body composition features. Particularly, the participants with a greater aerobic power presented a higher percentage of muscle mass and a lower percentage of FM. Bioelectrical PhA and FM were identified as valid predictors of VO_2max_, regardless of body mass and age. To date, this is the first study to investigate these relations in futsal players, suggesting that BIVA can be used as a tool to evaluate aerobic power.

BIVA showed that the participants with a lower VO_2max_ (T1 group) presented a shorter mean vector than the futsal players with a higher VO_2max_ (T3 group). The vector length is inversely related with TBW that is determined by the amount of intra and extra cellular fluids [21]. The shorter vector of the T1 group may be due to a higher TBW with a lower ICW/ECW ratio. Indeed, PhA, calculated as the arctangent between Xc and R, is directly related to the ICW/ECW ratio [20]. In the participants with similar R/H, the low PhA was also due to a lower Xc/H, which is known to be negatively associated with ECW [28] and representative of the integrity of cell membranes and tissue interfaces [29]. In healthy athletes not affected by acute or chronic fatigue, a high PhA could be the consequence of a high percentage of muscle mass and a low percentage of FM [30], as it turned out in the present study. In fact, a high level of FM may lead to a fluid retention into the extracellular spaces and in some cases was stated that generate inflammation [31,32]. Conversely, a high level of muscle mass encompasses a fluid balance in favor of the intracellular compartment [30]. A shorter and rightward vector may then represent a situation of higher FM% and lower PhA, both values that negatively affect aerobic power.

The results of the present study identified FM and PhA as valid predictors of VO_2max_, even when adjusted for body mass and age. Previous studies are in line with these results reporting the negative effect of FM on aerobic power [14,33,34]. However, while it was suggested that FFM is positively associated with and enhanced aerobic power [14], in the present study the muscle mass, one of the major components of the FFM, did not result among the VO_2max_ predictors. It could be speculated that over a certain threshold, increases in muscle mass do not lead to VO_2max_ gains, as already suggested by a previous study on combat athletes [35]. Several studies investigated the role of body composition in determining aerobic fitness [13,14,34]. However, all of them considered only FM and FFM. The quantification of FFM preclude the evaluations of several body components and their distribution among the body. For example, different distributions of ICW and ECW can result in a similar TBW. In this regard, the body fluid distribution reflected by PhA was identified as a VO_2max_ determinant in the present study. Therefore, evaluating FFM without considering its sub-components may result in a poor assessment of body composition. The negative influence of FM on flexibility and mobility has already been reported in previous findings [33,36]. Additionally, PhA associations with anaerobic performance, such as sprints, and aerobic outcomes like running time in endurance competitions have been described in the literature [37,38]. Therefore, practitioners should consider reducing FM and increasing PhA in order to achieve a greater aerobic power. Both these adaptations can be achieved through nutritional and training interventions [22,31]. In contrast, muscle mass does not seem to influence fitness levels in futsal players. However, body composition may play only a moderate part in the prediction of aerobic power. Therefore, physiological characteristics such as ventilatory functions as well as biomechanical and technical features involved in the running economy should also be considered across the competitive season in futsal players [11].

Previous research studies showed that a leftward vector displacement was associated with an improvement in aerobic and anaerobic performance [39,40]. In particular, vector shifting from the right to the left side and from the bottom to the upper part of the R-Xc graph occurred after a training macrocycle in swimmers [40]. This also results in a FM reduction and in an aerobic performance improvement [28]. Therefore, monitoring vector displacements may provide important insights related to changes in body composition and fitness levels. In this regard, reference zones among the R-Xc graph were proposed for the athletic population, considering the first phase of the season when a high fitness level should be achieved after the preparatory phase [26]. However, these target zones have been identified in athletes involved in several different sports and may not be used to just characterize futsal players. Also, reference percentiles for athletes have been proposed [41], identifying an average value of 7.7 degree around the 50th percentile. In this study, to achieve these BIA-related standards, the athletes included in the T1 group should reduce FM and ECW in order to move the vector to the left side of the R-Xc graph. On the contrary, T3 athletes should increase PhA to induce a shortening and a leftward displacement of the vector. The futsal players with a higher VO_2max_ of our study presented lower values than previous data reported in literature on elite futsal players [8]. This may be due to the different competitive phases evaluated in these studies. In fact, the participants of the present study were assessed during the preparatory phase where physical adaptations are not achieved yet. Further studies should assess if vector displacements to defined target zone and PhA increases would results in a VO_2max_ improvement.

The strength of the present study resides in the reference procedures used for determining VO_2max_, body composition, and bioelectrical parameters. In fact, breath-by-breath devices, DXA, and BIA are among the most accurate methods to quantify VO_2max_ and fat, estimate muscle mass, and detect bioelectrical phase angle, respectively. Although more body composition parameters such as TBW, ECW, and ICW would have been estimated with BIA, we preferred to avoid the use of BIA-based predictive equations since no specific formulas for futsal players are available in literature to date [18], therefore this non-specificity would have resulted in estimation errors. In this regard, the lack of dilution techniques as reference procedures for determining body fluids should be recognized as a limitation. Additionally, DXA cannot be assumed as the state-of-the-art method for evaluating FM, but it is employed in conjunction with air plethysmography and dilution techniques in the four-compartment molecular model, which is currently considered the gold standard procedure [18,42]. In the same way, magnetic resonance would be used for quantifying muscle mass instead of DXA. Furthermore, the results of this study cannot be generalized, but could be applicable to assessments performed with foot-to-hand technology at a single frequency of 50 kHz, since differences in the analysis were highlighted between bioelectrical parameters measured using different type of devices [43,44].

## 5. Conclusions

Body fat and bioelectrical phase angle can be considered as valid predictors of VO_2max_ in professional futsal players. In contrast, muscle mass is not associated with aerobic power. Bioelectrical impedance vectors positioned on the lower pole of the R-Xc graph were found in futsal players with a lower VO_2max_. Using BIVA, the length of the vector reflects the body fluid content, while phase angle is informative of their distributions among the intra and extra cellular spaces. Inferior aerobic power was found in athletes with higher TBW, lower phase angle and higher body fat, regardless of their muscle mass. BIVA seems to be a feasible field method for assessing body composition while avoiding the use of prediction equations.

## Figures and Tables

**Table 1:** Body composition characteristics (mean ± standard deviation) for the athletes grouped by tertiles of VO_2max_ and results of the analysis of variance (ANOVA) are shown.

Variable	T1, n = 16 (V0_2max_ = 42.9 ± 3.7 mL/kg/min)	T2, n = 16 (VO_2max_ = 50.1 ± 1.7 mL/kg/min)	T3, n = 16 (VO_2max_ = 58.2 ± 3.9 mL/kg/min)	ANOVA
Body mass (kg)	81.0 ± 0.6 ^2,3^	71.5 ± 7.5 ^1,3^	66.2 ± 5.4 ^1,2^	F = 15.5, *p* < 0.001
BMI (kg/m^2^)	24.3 ± 3.1 ^3^	23.3 ± 1.5	22.0 ± 1.6 ^1^	F = 4.64, *p* = 0.015
DXA				
Muscle mass (kg)	33.3 ± 3.6	31.2 ± 4.2	31.8 ± 4.2	F = 1.1, *p* = 0.349
Muscle mass (%)	41.6 ± 6.8 ^3^	43.8 ± 3.9	48.3 ± 7.5 ^1^	F = 4.7, *p* = 0.014
Fat mass (kg)	17.9 ± 6.0 ^2,3^	12.8 ± 3.0 ^1,3^	9.8 ± 2.1 ^1,2^	F = 16.1, *p* < 0.001
Fat mass (%)	22.1 ± 5.5 ^2,3^	18.2 ± 3.3 ^1,3^	15.2 ± 2.5 ^1,2^	F = 11.8, *p* < 0.001
BIA				
R/H (ohm/m)	279.3 ± 27.3	282.9 ± 18.5	293.4 ± 22.3	F = 1.6, *p* = 0.212
Xc/H (ohm/m)	33.7 ± 3.0 ^3^	35.5 ± 3.0	38.4 ± 3.3 ^1^	F = 9.3, *p* < 0.001
Phase angle (degree)	6.9 ± 0.4 ^3^	7.2 ± 0.4	7.5 ± 0.5 ^1^	F = 7.7, *p* = 0.001

Note: BMI = body mass index, DXA = dual-energy X-ray absorptiometry, BIA = bioelectrical impedance analysis, R/H = resistance standardized for height, Xc/H = reactance standardized for height. ^1^ = different (*p* < 0.016) from T1; ^2^ = different from T2; ^3^ = different from T3.

**Table 2:** Adjusted models using fat and muscle mass, and phase angle as independent variables for determining VO_2max_.

Model	R^2^	Std. Error	β	95% CI	*p*-Value
Fat mass					
Model ^a^	0.453	0.23	−0.335	−0.94, −0.02	0.046
Muscle mass					
Model ^a^	0.410	0.15	0.120	−0.21, 0.64	0.371
Phase angle					
Model ^a^	0.511	1.64	0.351	1.95, 8.54	0.003

Abbreviations: R^2^, coefficient of determination; β, Unstandardized coefficients beta; CI, confidence interval; Std. Error, standard error; VO_2max_, maximum rate of oxygen consumption. ^a^, adjusted for age and body mass.

## Data Availability

Data can be obtained from the corresponding author on francesco.campa3@unibo.it.
